# Pre-Stenting: Is Smaller Better? A Multicentric Evaluation of 4.8 F and 6 F JJ Stents in Retrograde Intrarenal Surgery

**DOI:** 10.3390/medicina62040778

**Published:** 2026-04-17

**Authors:** Erdinc Dincer, Orkunt Ozkaptan, Cengiz Canakci, Mehmet Gurkan Arikan, Burak Citamak, Yusuf Diker, Osman Murat Ipek, Kubilay Can Caglar, Alkan Cubuk

**Affiliations:** 1 Department of Urology, Health Sciencies University, Kartal Dr. Lutfi Kirdar City Hospital, Istanbul 34865, Türkiye; ozkaptanorkunt@gmail.com (O.O.); cengizcanakci@hotmail.com (C.C.); omipek@hotmail.com (O.M.I.); drkubilaycancaglar@gmail.com (K.C.C.); 2 Department of Urology, Faculty of Medicine, Cukurova University, Adana 01330, Türkiye; mgarikan26@gmail.com; 3 Department of Urology, Acibadem Atakent Hospital, Istanbul 34307, Türkiye; burakcitamak@gmail.com; 4 Department of Urology, Iskenderun Palmiye Hospital, Hatay 31200, Türkiye; ydiker@gmail.com; 5 Department of Urology, Bursa Medicana Hospital, Bursa 16110, Türkiye; alkancubuk@hotmail.com

**Keywords:** preoperative JJ stent, diameter, retrograde intrarenal surgery, urinary symptoms, complications

## Abstract

*Background and Objectives*: In this study, we aimed to compare the effects of 4.8 F and 6 F JJ stents on irritative symptoms and success rate in patients with a preoperative JJ stent prior to RIRS. *Materials and Methods*: A total of 316 patients who underwent JJ stent implantation prior to retrograde intrarenal surgery were retrospectively analyzed. Patients were classified into Group 1 (4.8 F stent) and Group 2 (6 F stent) based on stent diameter. Urinary symptoms prior to the insertion of the JJ stent and the day before RIRS were evaluated using the Quality of Life (QoL) score (VAS), International Prostate Symptom Score (IPSS), and Visual Analog Scale. The groups were compared in terms of demographic data, stone characteristics, perioperative data, length of stay, complications, stone-free rates (SFRs) and change in total VAS score, IPSS and QoL. *Results*: The patients’ demographic data and the stone characteristics of the two groups were similar. There was no statistical difference between groups in the SFR, length of stay, or total complication rates (*p* = 0.5, *p* = 0.159, and *p* = 0.13, respectively). Operation time was similar between the groups (*p* = 0.13). Although the change in total VAS scores was similar between the two groups (*p* = 0.1), QoL was higher in Group 1 compared with Group 2 (*p* < 0.001). Group 2 demonstrated worse outcomes in total IPSS, as well as in the storage subscores (Q2, Q4, and Q7) and voiding symptom subscores (Q1, Q3, Q5, and Q6), with all comparisons showing statistical significance (*p* < 0.001 for each). *Conclusions*: Varying calibers of JJ stents have comparable success rates and complication outcomes. However, when the stent caliber is reduced, patients experience better QoL and fewer lower urinary tract symptoms. Smaller-diameter stents were associated with significantly better symptom-related outcomes without compromising surgical success.

## 1. Introduction

Retrograde intrarenal surgery (RIRS) is one of the main surgical choices for treating 10–20 mm kidney stones with decreasing hemorrhage and length of stay [1]. The RIRS technique has been developed following the first flexible ureterorenoscope (f-URS) described by Marshall in 1964 [2]. The ureteral access sheath (UAS), first described in 1974, has been increasingly adopted by urologists as a routine adjunct in RIRS. It facilitates access to the kidney during the procedure and is considered to provide several advantages, including reduction in intrarenal pressure (thereby potentially decreasing the risk of infection), improvement of endoscopic visualization, and enhancement of stone fragmentation efficiency [3]. UAS insertion can sometimes be prevented by an orifice or ureteral strictures, even though minimally invasive surgery is becoming more common. Waseda et al. reported that the failure rate of UAS insertion can reach up to 20% [4]. Moreover, the use of UAS may reduce ureteral blood flow, potentially leading to stricture formation in the long term. In clinical routine, a JJ stent is placed in cases where the UAS cannot be placed to facilitate passive dilatation, prevent ureteral trauma, and ease subsequent UAS placement [5].

The impact of existence of JJ stent prior to surgery has been evaluated before. Studies have shown that inserting in a JJ stent before surgery makes it easier to access the collecting system during ureteroscopy, in turn leading to a higher stone-free rate (SFR) [6]. Bai et al. compared two groups of patients: those who received a 7 F stent prior to RIRS and those who did not. In the pre-stented group, operation time was lower and SFR was found to be higher at the first postoperative month [7].

Although JJ stents have certain benefits, they may cause irritative symptoms in patients such as frequency, urgency, dysuria, flank or suprapubic pain, and hematuria due to bladder irritation [8,9]. Studies have shown that as the diameter of the ureteral stent increases, irritative symptoms tend to become more pronounced [10,11,12], but the outcomes of the presence of a JJ stent prior to RIRS remain controversial. Although the presence of a preoperative ureteral stent has been shown to facilitate access and potentially improve surgical outcomes, the role of stent diameter remains unclear. Existing studies have primarily focused on either surgical outcomes or stent-related symptoms, and the available evidence regarding the effect of stent diameter is limited and inconsistent. More importantly, no previous study has evaluated the impact of different stent diameters on both RIRS outcomes and patient-reported symptoms under standardized procedural conditions. This study aimed to evaluate the clinical relevance of preoperative ureteral stent diameter in patients undergoing RIRS by comparing 4.8 F and 6 F JJ stents in terms of stent-related symptoms and operative success rates.

## 2. Material and Methods

This research was conducted in accordance with the Declaration of Helsinki and permission was obtained from the local Institutional Ethics Committee. (Decision No: 2025/10.099/19/29, Decision Date: 27 August 2025). A retrospective analysis was performed using prospectively collected data. The study population comprised patients who had received JJ stent implantation before undergoing RIRS. The indications for patient pre-stenting were renal colic, complicated urinary tract infection, or unsuccessful UAS insertion in prior surgery.

In this multicentric cohort study, we evaulated a total of 316 patients (*n* = 201, 64% male; *n* = 115, 36% female) aged over 18 years who underwent RIRS due to a renal stone measuring between 10 and 20 mm in diameter between January 2022 and July 2025 at three high-volume urology centers were evaluated, and all procedures were performed by five experienced urologists.

Inclusion criteria were as follows: diagnosis of a single renal stone measuring between 10 and 20 mm in diameter, absence of anatomic anomaly (ureteropelvic junction (UPJ) obstruction, rotational anomaly, solitary kidney, ectopic kidney, horseshoe kidney, etc.), and no prior history of renal or ureteral stone surgery. We included only patients with a single renal stone measuring 10–20 mm in diameter to ensure a homogeneous study population and reduce potential bias.

Patients were excluded if they had undergone ureteral stricture surgery or had undergone bilateral stent insertion, received pelvic or abdominal radiation, or if their preoperative or postoperative clinical or radiological data were absent. Because the literature indicates that stent position may affect lower urinary tract symptoms (LUTS), we eliminated individuals with ureteral stents traversing the bladder midline [9,13,14]. Patients receiving active medical treatment for lower urinary tract symptoms (including α-blockers, 5-alpha reductase inhibitors, and antimuscarinic agents), and those with a history of neurogenic bladder were excluded from the study.

### 2.1. Study Design, Clinical Data and Parameters

Patients were divided into two groups based on the JJ stent diameter placed prior to RIRS: 210 patients with 4.8 F stent (Group 1) and 106 with 6 F stent (Group 2). The selection of stent diameter (4.8 vs. 6 Fr) was not randomized and was based on the surgeon’s or center’s routine practice rather than patient- or procedure-specific factors. Furthermore, a predefined protocol for determining stent diameter was not applied. Patients’ baseline characteristics, stone size, operation time and complications were recorded. On the day before insertion of the JJ stent and the day before RIRS, the Visual Analog Scale (VAS), International Prostate Symptom Score (IPSS) and Quality of Life Score (QoL) were evaluated to assess stent-related symptoms. In all patients, the duration of stent dwelling was 14 days. RIRS was performed after the urine culture was sterile. SFR was defined as the absence of residual stones or the presence of residual fragments smaller than 4 mm following treatment [15]. SFR was evaluated with non-contrast enhanced computed tomography at the first postoperative month. Any additional interventions required within 90 days postoperatively due to residual or recurrent stones were classified as secondary surgical interventions. At the end of the procedure, patients were re-stented with a conventional JJ stent (without extraction string) of the same diameter as the one previously in place, which was then removed with cystoscopy at 2 weeks after RIRS.

### 2.2. RIRS Technique

All patients received a second-generation cefazolin for antibiotic prophylaxis one hour before surgery, and all procedures were performed under general anesthesia in the supine lithotomy position. Following removal of the preexisting JJ stent, a 0.035-inch polytetrafluoroethylene-coated guidewire (Sensor^®^, Boston Scientific, Marlborough, MA, USA) was inserted into the renal pelvis. An 8/9.8 F semirigid ureteroscope (Richard Wolf, Knittlingen, Germany or Karl-Storz, Tuttlingen, Germany) was used to evaluate the entire ureter for the presence of ureteral strictures. After placement of a non-suction conventional 9.5–11.5 F ureteral access sheath (UAS) (The CookFlexor, Cook Medical, Bloomington, IN, USA), a disposable flexible ureterorenoscope (Olympus, Tokyo, Japan or Hugemed, Shenszhen, China) was introduced into the collecting system, where the stone was visualized.

A Holmium: YAG laser with a 272 µ fiber was employed for stone fragmentation (energy level of 0.5–1.2 J and a pulse rate of 10–18 Hz) (30 W LISA Shpinx Jr, LISA Laser Products GmbH, Katlenburg-Lindau, Germany or 30 W Litho Laser, Quanta, Quanta System S.p.A., Samarate, Italy). Stone fragments larger than 4 mm were retrieved using a 2.2 F nitinol stone basket. Upon completion of the operation, a JJ stent of the same diameter as the preoperative stent was reinserted.

### 2.3. Statistics

The Kolmogorov–Smirnov test, skewness and kurtosis values were employed to assess the normality of the distribution. Descriptive statistics for continuous variables were presented as mean  ±  standard deviation or median (25th percentile–75th percentile) according to the distribution type, whereas categorical data were conveyed as frequency (%). The baseline patient characteristics, initial scores on the IPSS and QOL prior to ureteral stent implantation, and the mean change from baseline in the IPSS and QOL were evaluated between the two groups utilizing *t* tests or χ^2^ tests. A multivariable logistic regression analysis was conducted to identify characteristics that are independently linked with SFR, including clinically significant variables such as stone size, stone location, stone density, and JJ stent size. The multivariable model included variables with a *p*-value < 0.05 from the univariate analysis. The odds ratios (ORs) and 95% confidence intervals (CIs) were calculated. Analyses were conducted using IBM SPSS version 25.0 (IBM Corp., Armonk, NY, USA) and two-tailed *p*-values of less than 0.05 were deemed statistically significant.

## 3. Results

The investigation involved a total of 316 participants, with Group 1 comprising 210 patients, and Group 2 comprising 106. The baseline features of the patients in the two study groups were comparable concerning sex, age, BMI, stone location, stone size, stone density, and IPSS [Table 1].

Table 2 presents a comparative analysis of the changes from baseline in the IPSS and Quality of Life between the two groups. People in Group 2 had worse overall International Prostate Symptom Score (IPSSs) (*p* = <0.001) compared to those in Group 1. This was also true for the subscores for intermittency (*p* = <0.001), urgency (*p* = <0.001), frequency (*p* = 0.00), and voiding (Q1 + Q3 + Q5 + Q6; *p* = <0.001) and storage symptoms (Q2 + Q4 + Q7; *p* = <0.001). The change in QOL score from baseline was statistically higher in Group 2 than in Group 1 (2.17 ± 0.97 vs. 2.91 ± 1.25; *p* = <0.001), while neither group showed any significant difference in VAS score (3.72 ± 1.54 for Group 1 vs. 4 ± 1.09 for Group 2; *p* = 0.1). The mean difference in total IPSS change between Group 2 and Group 1 was 4.08 (95% CI: 2.92 to 5.24), the mean difference in QoL score change was 0.74 (95% CI: 0.47 to 1.01), and for the VAS score change, the mean difference was 0.28 (95% CI: −0.02 to 0.58).

The SFR demonstrated similarity across the groups (162 (77.14%) vs. 86 (81.13%); *p* = 0.5). The operation time was similar between the groups (respectively, 52.62 ± 20.79 vs. 49.73 ± 12.87; *p* = 0.13), while hospitalization time indicated no significant difference between them. A multivariable logistic regression analysis was conducted to determine characteristics independently associated with SFR [Table 3]; no significant association was found. Stone location (OR = 1.218, 95% CI: 0.937–1.582, *p* = 0.140), stone density (OR = 1.000, 95% CI: 0.999–1.002, *p* = 0.498), or stent size (OR = 1.366, 95% CI: 0.677–2.756, *p* = 0.384). The size of the stone exhibited a marginal correlation with stone-free status (OR = 1.075, 95% CI: 1.002–1.153, *p* = 0.052), although it did not attain statistical significance at the established level.

Overall complication rates, as well as the rates of major and minor complications according to the Clavien–Dindo classification system, revealed no significant differences between the groups (*p* = 0.13 vs. *p* = 0.48, respectively). In Groups 1 and 2, nineteen patients and fourteen patients had minimal hematuria. Minimal hematuria was defined as temporarily macroscopic hematuria lasting less than 6 h and requiring neither transfusion nor transurethral irrigation. Ureteral mucosal injury was observed in ten patients in Group 1 and eight in Group 2, while postoperative infective complications needing pharmacological treatment were observed in eight patients in group 1 and seven in group 2.

## 4. Discussion

In this retrospective multicentric study, we evaluated the effectiveness of JJ stents of varying diameters inserted prior to RIRS. This study is, to our knowledge, the first investigation of the effects of varying stent diameters on both the success rate and stent-related symptoms. The primary finding of this multicentric study is that JJ stent diameter did not significantly influence surgical outcomes, specifically SFR, operation time, and overall complication rates, provided that a standardized-size UAS was utilized. These results suggest that increasing the stent caliber does not yield clinically meaningful improvements in surgical efficacy, thereby allowing surgeons to prioritize patient comfort without compromising procedural success.

Ureteral stents are necessary for endourological surgery, but they can cause irritation symptoms like hematuria and urinary tract symptoms like pain, pollakiuria, and dysuria. This is mostly because they cause bladder mucosal irritation, ureteral spasms, inflammation, and urinary reflux [12]. It has been reported that 80% of patients with stents experience unpleasant and painful symptoms, 32% report sexual dysfunction, and 58% suffer from work-life disturbances [16,17]. Ehsanullah et al. stated that smaller diameter offers benefits with lower LUTS and postoperative pain [11]. Similarly, Boom So Kin et al. also reported lower Ureteral Stent Symptom Questionnaire (USSQ) scores associated with smaller-diameter stents [10]. Taguchi et al. conducted a study assessing how 4.7 F and 6 F stents differentially affected LUTS and pain in patients who were prestented before URS [9]. They stated that urgency, intermittency, voiding symptoms, and total overactive bladder symptom scores (OABSS) were found to be lower in the 4.8 F group, while operation time and overall complication rates were similar [9]. However, they did not compare the SFR between groups [9].

In the present study, in patients prestented with a 4.8 F JJ stent, the change in total IPSS was smaller and the QoL scores were better compared with the 6 F prestented group. When we analyzed IPSS subscores, the 4.8 F stent provided greater benefit in both voiding and storage symptom domains. We attribute the improvement to the smaller diameter stent being softer, more flexible, and able to occupy a better position within the urinary system, thereby reducing irritation. Lennon et al. similarly demonstrated that the flexibility of the stent plays a role in the severity of stent-related symptoms [18]. In our study, while the change in VAS score did not differ between the groups, QoL was superior with the smaller diameter stent. From a patient-centered perspective, smaller-diameter stents may be preferable, as they improve quality of life and reduce lower urinary tract symptoms without compromising surgical success.

The effect of the presence of a preoperative JJ stent on SFR has been the subject of previous research. In 2022, Diatmika et al. published a review comparing different stent diameters in pre-stented patients. The review evaluated three studies comparing stents smaller than 5 F with 6 F stents in pre-stented patients and identified no significant differences between the groups regarding VAS scores or stone-free rates [12]. Nestler et al. compared three different stent diameters (4.7 F, 6 F, and 7 F) in pre-stented patients before URS and stated that the success of surgery was sufficient in all three groups (above 82%, *p* > 0.15) [19]. Çubuk et al. similarly found no correlation between stent diameter and SFR [20]. In both studies, patients who underwent URS were selected. Recently, Sahin et al. evaluated the impact of preoperative JJ stent diameter on RIRS outcomes. They stated that the presence of a preoperative 4.8 F or 6 F JJ stent had no effect on overall UAS insertion success, complication rates, or stone-free rates. However, in the group of patients with a 6 F stent, the rate of placement of a larger UAS was higher, which improved drainage flow and visualization quality, thereby shortening the operative time [21]. In the present study, we found that size difference in preoperative JJ stent diameter did not have a significant impact on the overall surgical success in patients who underwent RIRS. This may be due to the comparable SFR across both groups from similar stone parameters in all patients and the standardized use of single-sized UAS.

Certain postoperative complications such as fever, hematuria and ureteric injury may arise following RIRS. Numerous studies have clearly documented these complications and suggest that pre-stenting may play a significant role in reducing their incidence. Chai et al. reported that both infectious and overall complication rates were lower in pre-stented patients compared with non-stented groups (13.62% and 15.89%, respectively) [22]. Nonetheless, they indicated no statistical difference across the complication subgroups, specifically hematuria, fever, and sepsis [22]. Similarly, Senel et al. observed higher rates of intraoperative complications in the non-stented group (19.2% in the pre-stented and 28.7% in the non-stented group) [23]. On the other hand, they reported that comparison of the postoperative complication rates among the study groups revealed no statistically significant differences [23]. Nevertheless, neither of these studies provided data regarding stent diameter. Our analysis demonstrated that varying stent diameters were not associated with statistically significant differences in overall complication rates. This may be explained by the use of a standardized UAS size (9.5–11.5 F), which minimizes variability in intrarenal pressure, irrigation efficiency, and visualization—key determinants of surgical outcomes. In contrast, Şahin et al. reported the use of larger UAS sizes in the 6 F group; however, no significant differences were observed between groups in terms of complication rates, further suggesting that stent diameter alone may not be a decisive factor in overall complications [21].

Several studies have investigated whether stent diameter influences operation time. Taguchi et al. reported that variations in stent diameter did not have a significant effect on operative duration [9]. Similarly, in their comparative study of 4.8 F and 6 F stents, Çubuk et al. demonstrated that stent diameter was not associated with differences in total operation time [20]. In the present study, consistent with the existing literature, we found that pre-stenting with different stent diameters prior to RIRS did not have a significant effect on operation time. In our investigation, a single-sized UAS (9.5–11.5 F) was employed, and no significant changes were seen across groups for stone parameters. In one of the largest population-based studies on pre-stenting published by Şahin et al., it was reported that operation time was shorter in patients with a 6 F pre-stented ureter. They also noted that a larger UAS diameter could be used in the 6 F group. They attributed the shorter operation time by improved visualization and increased drainage flow to the use of a larger UAS in the patients with larger stent diameter [21].

This study has several strengths. First, to the best of our knowledge, this is the first study to investigate the impact of different ureteral stent diameters on both RIRS outcomes and stent-related symptoms. In addition, the study is strengthened by its relatively large sample size and multicentric design, as well as the inclusion of a homogeneous patient population with standardized stone characteristics. In order to minimize confounding factors and ensure a homogeneous study population, only patients with a single renal stone measuring between 10 and 20 mm were included. By restricting the study population, we aimed to better isolate the specific impact of stent diameter on surgical outcomes and stent-related symptoms. Furthermore, the use of a uniform ureteral access sheath (UAS) size across all cases represents a key methodological strength, allowing for a more precise evaluation of the independent effect of stent diameter. These factors enhance the generalizability of the findings and strengthen the validity of the conclusions.

This study carries several limitations that warrant consideration. To begin with, its retrospective design restricts the ability to control for confounding factors and may introduce bias stemming from incomplete or imprecise data. The involvement of multiple surgeons, rather than a single operator, may have influenced the SFR outcomes even if all the surgeons had sufficient experience. Additionally, the choice of stent diameter was not randomized and was left to the surgeon’s discretion, which we acknowledge as a potential source of selection bias, despite comparable baseline parameters being similar between the groups. Despite employing IPSS, VAS, and QoL ratings, the literature indicates that the USSQ is the gold standard for assessing stent-related symptoms, which constitutes a notable limitation of our investigation. IPSS and VAS were used in this study due to their practicality and routine clinical applicability. However, these tools may not comprehensively capture all domains of stent-related symptoms, which represents a limitation.

## 5. Conclusions

We found that varying stent diameters resulted in comparable outcomes regarding SFR and ureteral injury. However, smaller-caliber stents were associated with more favorable outcomes regarding QoL and lower urinary tract symptoms. Our findings suggest that smaller-caliber stents may be preferred in the preoperative setting before endourologic stone treatment, as they improve patient comfort without compromising surgical outcomes. This has direct implications for clinical practice and may help guide more patient-centered decision-making. Future prospective, randomized studies are needed to further improve these findings and to explore the combined effects of stent diameter, material, and positioning on both surgical outcomes and patient-reported symptoms using standardized tools such as the USSQ.

## Figures and Tables

**Table 1:** Comparative analysis of clinical and stone-related characteristics between two patient populations.

	Group 1 (4.8 F)	Group 2 (6 F)	*p*
Number of cases (*n*, %)	210 (66.45%)	106 (33.54%)	
Age (mean ± SD)	48.47 ± 14.55	45.25 ± 13.01	0.055
Gender (*n*, %)			0.47
Male	137 (65.23%)	64 (60.38%)	
Female	72 (34.28%)	42 (39.62%)	
BMI (kg/m^2^) (mean ± SD)	27.4 ± 4.6	26.9 ± 4.5	0.79
CCI (*n*, %)			0.28
0–1	142 (67.1%)	76 (73.6%)	
≥2	68 (32.9%)	28 (26.4%)	
Preoperative serum creatinine level (mg/dL) (mean ± SD)	0.98 ± 0.39	0.93 ± 0.24	0.85
Stone location (*n*, %)			0.53
Upper calyx	6 (2.8%)	3 (2.83%)	
Middle calyx	25 (11.9%)	9 (8.5%)	
Lower calyx	33 (15.7%)	18 (17%)	
Renal pelvis	75 (35.7%)	32 (30.1%)	
Upper ureter	71 (33.8%)	46 (43.4%)	
Stone density (Hounsfield Unit (mean ± SD)	947.81 ± 311.04	940.65 ± 182.66	0.827
Stone size (mm) (mean ± SD)	13.18 ± 4.71	12.49 ± 4.66	0.22
Postoperative serum creatinine level (mg/dL) (mean ± SD)	0.97 ± 0.49	1.05 ± 0.87	0.31
Baseline IPSS			
Q1: Incomplete emptying	1.1 ± 0.9	1.2 ± 1.0	0.28
Q2: Frequency	1.2 ± 0.9	1.3 ± 1.0	0.31
Q3: Intermittency	1.0 ± 0.8	1.1 ± 0.9	0.35
Q4: Urgency	1.2 ± 0.8	1.3 ± 1.0	0.29
Q5: Weak stream	1.1 ± 0.9	1.2 ± 0.9	0.33
Q6: Straining	0.8 ± 0.7	0.9 ± 0.7	0.30
Q7: Nocturia	1.3 ± 1.0	1.4 ± 1.1	0.27
Total IPSS	7.7 ± 3.0	8.3 ± 3.3	0.24
Baseline QoL	2.05 ± 1.12	2.28 ± 1.25	0.11
Stone location			0.67
Right	90 (42.9%)	42 (39.6%)	
Left	120 (57.1%)	64 (60.3%)	

BMI = Body mass index, CCI = Charlson Comorbidity Index.

**Table 2:** Comparison of perioperative outcomes and alterations from baseline in the International Prostate Symptom Score, Visual Analog Scale, and Quality of Life.

	Group 1 (4.8 F)	Group 2 (6 F)	*p*
Stone-free rate (*n*, %)	162 (77.14%)	86 (81.13%)	0.5
Operation time (min.) (mean ± SD)	52.62 ± 20.79	49.73 ± 12.87	0.13
Hospitalization time (day)			0.159
median, (IQR)	1.0 (1)	1.0 (1)	
Total complication rate (*n*, %)	18 (8.57%)	15 (14.1%)	0.13
IPSS Parameters	5.14 ± 4.82	9.22 ± 4.98	<0.001
Q1: Incomplete emptying	0.66 ± 0.71	0.75 ± 0.62	0.28
Q2: Frequency	0.85 ± 0.85	1.77 ± 1	<0.001
Q3: Intermittency	0.86 ± 0.85	1.94 ± 1.14	<0.001
Q4: Urgency	0.9 ± 0.89	1.81 ± 1.03	<0.001
Q5: Weak stream	0.41 ± 0.61	0.48 ± 0.5	0.27
Q6: Straining	0.46 ± 0.53	0.56 ± 0.6	0.140
Q7: Nocturia	1.01 ± 1	1.91 ± 0.98	<0.001
Voiding symptoms *	2.38 ± 1.98	3.73 ± 1.98	<0.001
Storage symptoms **	2.76 ± 2.69	5.49 ± 2.82	<0.001
VAS	3.72 ± 1.54	4 ± 1.09	0.1
QoL	2.17 ± 0.97	2.91 ± 1.25	<0.001

IPSS = International Prostate Symptom Score, QoL = Quality of life. * Voiding symptom subscores were calculated by summing scores for questions (Q) 1, 3, 5, and 6. VAS = Visual Analog Scale; QoL = Quality of Life. ** Storage symptom subscores were calculated by summing the scores for questions (Q) 2, 4, and 7.

**Table 3:** Variables independently associated with Stone-Free Status: Multivariable logistic regression analysis.

Variable	β Coefficient	Standard Error	*p*	Exp(B)	95% CI
Stone Location	0.197	0.133	0.140	1.218	0.937–1.582
Stone Density (HU)	0.000	0.001	0.498	1.000	0.999–1.002
Stone Size (mm)	0.072	0.036	0.052	1.075	1.002–1.153
Stent Diameter (F)	0.312	0.358	0.384	1.366	0.677–2.756
Constant	−2.491	0.746	0.083		

Exp(B) = Odds Ratio; a *p*-value < 0.05 was considered statistically significant.

## Data Availability

The data that support the findings of this study are not publicly available due to them containing information that could compromise the privacy of research participants, but are available from the corresponding author [E.D., e-mail: drerdincdincer@gmail.com] upon reasonable request.

## References

[1] Türk C., Petřík A., Sarica K., Seitz C., Skolarikos A., Straub M., Knoll T. (2016). EAU Guidelines on Interventional Treatment for Urolithiasis. Eur. Urol..

[2] Jeong J.Y., Cho K.S., Jun D.Y., Moon Y.J., Kang D.H., Jung H.D., Lee J.Y. (2023). Impact of Preoperative Ureteral Stenting in Retrograde Intrarenal Surgery for Urolithiasis. Medicina.

[3] Lin C.B., Chuang S.H., Shih H.J., Pan Y. (2024). Utilization of Ureteral Access Sheath in Retrograde Intrarenal Surgery: A Systematic Review and Meta-Analysis. Medicina.

[4] Waseda Y., Takazawa R., Kobayashi M., Fuse H., Tamiya T. (2023). Different failure rates of insertion of 10/12-Fr ureteral access sheaths during retrograde intrarenal surgery in patients with and without stones. Investig. Clin. Urol..

[5] Yuk H.D., Park J., Cho S.Y., Sung L.H., Jeong C.W. (2020). The effect of preoperative ureteral stenting in retrograde Intrarenal surgery: A multicenter, propensity score-matched study. BMC Urol..

[6] Lumma P.P., Schneider P., Strauss A., Plothe K.D., Thelen P., Ringert R.H., Loertzer H. (2013). Impact of ureteral stenting prior to ureterorenoscopy on stone-free rates and complications. World J. Urol..

[7] Bai P., Wang T., Huang H., Wu Z., Wang X.G., Qin J.X., Wang H.Q., Chen B., Hu M.B., Xing J.C. (2021). Effect of Preoperative Double-J Ureteral Stenting before Flexible Ureterorenoscopy on Stone-free Rates and Complications. Curr. Med. Sci..

[8] Polat M.E., Karaaslan M., Yilmaz M., Olcucuoglu E., Sirin M.E. (2024). The effect of ureteral double J stent insertion on work performance in patients undergoing endoscopic stone treatment. Cent. Eur. J. Urol..

[9] Taguchi M., Yoshida K., Sugi M., Matsuda T., Kinoshita H. (2017). A ureteral stent crossing the bladder midline leads to worse urinary symptoms. Cent. Eur. J. Urol..

[10] Kim B.S., Choi J.Y., Jung W. (2020). Does a Ureteral Stent with a Smaller Diameter Reduce Stent-Related Bladder Irritation? A Single-Blind, Randomized, Controlled, Multicenter Study. J. Endourol..

[11] Ehsanullah S.A., Bruce A., Juman C., Krishan A., Krishan A., Higginbottom J., Khashaba S., Alnaib Z. (2022). Stent diameter and stent-related symptoms, does size matter? A systematic review and meta-analysis. Urol. Ann..

[12] Diatmika A.A.N.O., Djojodimedjo T., Kloping Y.P., Hidayatullah F., Soebadi M.A. (2022). Comparison of ureteral stent diameters on ureteral stent-related symptoms: A systematic review and meta-analysis. Turk. J. Urol..

[13] Rane A., Saleemi A., Cahill D., Sriprasad S., Shrotri N., Tiptaft R. (2001). Have stent related symptoms anything to do with placement technique?. J. Endourol..

[14] Giannarini G., Keeley F.X., Valent F., Manassero F., Mogorovich A., Autorino R., Selli C. (2010). Predictors of morbidity in patients with indwelling ureteric stents: Results of a prospective study using the validated Ureteric Stent Symptoms Questionnaire. BJU Int..

[15] Çavdar O.F., Aydin A., Tokas T., Tozsin A., Gadzhiev N., Sönmez M.G., Tekeli R., Ortner G., Kallidonis P., Akgül B. (2025). Residual stone fragments: Systematic review of definitions, diagnostic standards. World J. Urol..

[16] Bosio A., Alessandria E., Dalmasso E., Peretti D., Agosti S., Bisconti A., Destefanis P., Passera R., Gontero P. (2019). How bothersome double-J ureteral stents are after semirigid and flexible uretero scopy: A prospective single-institution observational study. World J. Urol..

[17] Mehra K., Manikandan R., Dorairajan L.N., Kodakkattil S.S., Kalra S. (2020). Effect of ureteral stent length and position of stent coil in blad der on stent-related symptoms and quality of life of patients. Cureus.

[18] Lennon G.M., Thornhill J.A., Sweeney P.A., Grainger R., McDermott T.E.D., Butler M.R. (1995). “Firm” versus “soft” double pigtail ureteric stents: A randomised blind comparative trial. Eur. Urol..

[19] Nestler S., Witte B., Schilchegger L., Jones J. (2020). Size does matter: Ureteral stents with a smaller diameter show advantages regard ing urinary symptoms, pain levels and general health. World J. Urol..

[20] Cubuk A., Yanaral F., Ozgor F., Savun M., Ozdemir H., Erbin A., Yuksel B., Sarilar O. (2018). Comparison of 4.8 Fr and 6 Fr ureteral stents on stent related symptoms following ureterore noscopy: A prospective randomized controlled trial. Kaohsiung J. Med. Sci..

[21] Şahin M.F., Özman O., Teke K., Şimşekoğlu M.F., Akgül M., Başataç C., Çınar Ö., Çakır H., Sıddıkoğlu D., Yazıcı C.M. (2025). The Impact of Preoperative JJ Stent Diameter on Retrograde Intrarenal Surgery: A RIRSearch Group Study. J. Laparoendosc. Adv. Surg. Tech. A.

[22] Chai C.A., Teoh Y.C., Tailly T., Emiliani E., Inoue T., Tanidir Y., Gadzhiev N., Bin Hamri S., Ong W.L., Shrestha A. (2023). Inferences from patients of the Global Multicentre Flexible Ureteroscopy Outcome Registry (FLEXOR). Minerva Urol. Nephrol..

[23] Senel S., Gultekin H., Kizilkan Y., Ozden C., Ceviz K., Koudonas A., Sevinc A.H. (2024). The role of preoperative ureteral stenting in retrograde intrarenal surgery outcomes for renal stones: A matched-pair analysis. Cent. Eur. J Urol..

